# Crystal Structure and Intermolecular Energy for Some Nandrolone Esters

**DOI:** 10.3390/molecules28207179

**Published:** 2023-10-19

**Authors:** Liviu Mare, Marieta Muresan-Pop, Pompilia Mioara Purcea Lopes, Alexandru Turza, Gheorghe Borodi, Violeta Popescu

**Affiliations:** 1 Physics & Chemistry Department, Technical University of Cluj-Napoca, 400114 Cluj-Napoca, Romania; mareliviumarius@gmail.com (L.M.); mioara.lopes@im.utcluj.ro (P.M.P.L.); violeta.popescu@chem.utcluj.ro (V.P.); 2 Interdisciplinary Research Institute in Bio-Nano-Sciences, Babes-Bolyai University, 42 Treboniu Laurian, 400271 Cluj-Napoca, Romania; mmuresanpop@gmail.com; 3 National Institute for R&D of Isotopic and Molecular Technologies, 67-103 Donat, 400293 Cluj-Napoca, Romania

**Keywords:** nandrolone, ester, crystal structure, intermolecular interaction, solubility

## Abstract

Nandrolone (Estr-4-en-17β-ol-3-one) is a derivative of testosterone and a naturally occurring anabolic–androgenic agent which belongs to the steroid group. Crystal structures of four short, medium and long esterified forms of nandrolone, including propionate, phenylpropionate, cypionate and undecanoate were determined using single-crystal X-ray diffraction. Crystal packing, supramolecular features and intermolecular interactions were described based on a quantitative and qualitative Hirshfeld surfaces analysis accompanied by evaluation of crystal energies and intermolecular interactions computation. Also, the solubility of the esters was investigated from a pharmaceutical perspective.

## 1. Introduction

Nandrolone or (Estr-4-en-17β-ol-3-one), also known as 19-nortestosterone, is chemically a testosterone derivative (demethylated at the C19 position) and a naturally occurring anabolic steroid, and is one of the most popular worldwide. It can be detected in the body in trace amounts [1] and it is synthesized during pregnancy [2].

Anabolic–androgenic steroids which include testosterone, its analogues and derivatives play a major role in the development of male reproductive tissues and promote the appearance of secondary male characteristics, including increased muscle mass, bone mass and density, aggression and body hair growth [3]. Nandrolone binds to the androgen receptor, further exerting the anabolic and androgenic properties in certain tissues specific to testosterone [4].

From a medical point of view, nandrolone is used to treat anemia, osteoporosis, and muscle wasting (cachexia) and to counteract catabolic conditions such as major burns, AIDS and cancer [5,6]. Being an agent which targets the androgen receptor, it provides increased protein synthesis and greatly improves skeletal muscle recovery, which makes it effective in uses for performance-enhancing purposes by athletes [5]. This practice is illegal and prohibited by the World Anti-Doping Agency.

**Figure 1:**
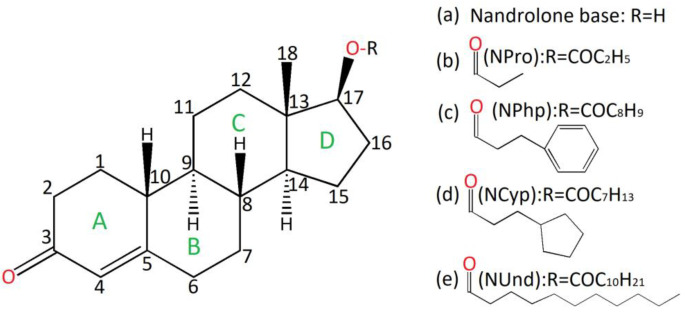
Chemical structure of nandrolone (Estr-4-en-17β-ol-3-one) illustrating the steroid skeleton numbering system (**a**) and the investigated nandrolone-based esters: propionate (**b**), phenylpropionate (**c**), cypionate (**d**) and undecanoate (**e**).

The oral form of nandrolone has been studied and it was shown to have a low potency of one-tenth compared with its subcutaneous administration [7]. Nandrolone (Figure 1a) possesses a short half-life (a few hours); it is advantageous to subject it to 17β esterification, which greatly prolongs the half-life through the intramuscular/subcutaneous route of administration, thus avoiding daily injections [8]. In general, the longer the ester (attached carbon chain), the longer the half-life of the prodrug.

This paper addresses and characterizes four short, medium and long esterified nandrolone prodrugs as follows:

(i) Nandrolone propionate (19-nortestosterone 17β-propionate, abbreviated NPro, Figure 1b).
(ii) Nandrolone phenylpropionate (19-nortestosterone 17β-phenylpropionate, abbreviated NPhp, Figure 1c).
(iii) Nandrolone cypionate (19-nortestosterone 17β-cyclopentanepropionate, abbreviated NCyp, Figure 1d).
(iv) Nandrolone undecanoate (19-nortestosterone 17β-undecanoate, abbreviated NUnd, Figure 1e).

The backbone labeling of the steroid is illustrated here, based on the established numbering for compounds belonging to the steroid class of compounds [9].

With this being a popular steroid, the literature accounts for several crystal structures of other nandrolone-based prodrugs: nandrolone monohydrate [10], nandrolone acetate [11], anhydrous nandrolone [12,13,14] and some nandrolone cocrystals [14]. This paper focusses on the structural insights of some nandrolone esters. The analysis was accomplished using experimental techniques (single-crystal X-ray diffraction, powder X-ray diffraction, FT-IR spectroscopy, DTA/TG thermal analysis) and was completed through an investigation of intermolecular interactions. This was accomplished using computational methods, including crystal lattice energies, intermolecular interactions (nature and strength) and Hirshfeld analysis.

It is known, from a pharmaceutical standpoint, that some pharmaceutically active ingredients are poorly soluble in water are highly lipophilic. Such agents are suitable for dissolution in lipid preparations [15]. Various drugs, including steroids and sex hormones (estradiol, progesterone, testosterone esters and their analogues/derivatives), and some vitamins are embedded in mixtures using oils as carriers [16,17]. The solubilities of esters in some lipid-based solutions were evaluated. 

We considered that, due to the worldwide popularity of this hormone, it would be useful to investigate and supplement the structural features and solubility of some nandrolone esters.

## 2. Results

### 2.1. Analysis of Crystal Structures

A comparison between experimental (Exp) powder X-ray diffraction patterns and simulated (Sim) patterns that were generated based on the CIF files is shown in Figure S1 (Supplementary Materials). High purity and good overall structural homogeneity can be determined based on the good match between the patterns; the investigated single crystals are a good representative for the bulk of all four samples. Some missing or more intense diffraction lines for NPro and NUnd show the preferred orientation of crystallites. Crystal data and the refinement details of the studied nandrolone esters are presented in Table 1.

#### 2.1.1. NPro (Nandrolone Propionate)

**Figure 2:**
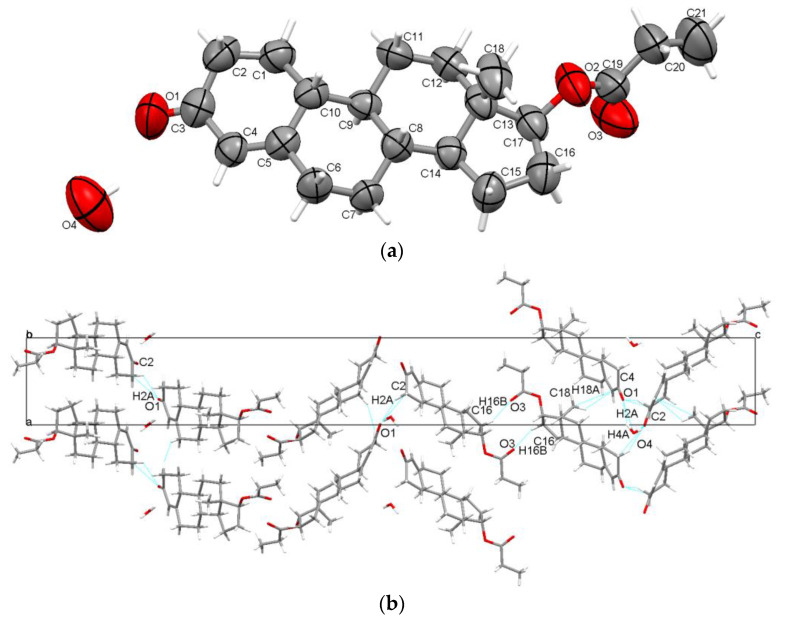
Displacement ellipsoid representations of asymmetric unit of TPro atoms at a 50% probability level (**a**); packing along the b-axis (**b**).

The propionate ester of nandrolone is one of the shortest available esters and it was found to crystallize in a trigonal system with an unusually long c axis (a = b = 7.761 Å, c = 56.211 Å) in the very rare P3_1_21 space group as a hemihydrate, with one steroid molecule and half a water molecule in the asymmetric unit (Figure 2a). The water molecule is situated at a symmetrical position, specifically at a 2-fold axis, and serves as a bridge between two O1 carbonyl oxygen atoms of steroid molecules through O4-H4A···O1 interactions, exhibiting an energy magnitude of −18.4 kJ/mol. The strengths of these interactions are outlined in Table S4 (Supplementary Materials). The carbonyl O1 oxygen is involved further in C2-H2A···O1 interactions with other steroid molecules (E_tot_ = −10.5 kJ/mol). The carbonyl O3 oxygen of the ester chain plays the role of acceptor in C16-H16B···O3 interactions, with an adjacent steroid molecule (E_tot_ = −31.9 kJ/mol). Further, the A ring is involved in the C-H···π interaction, being connected to the C18 methyl group (C18-H18A···C4 interaction with E_tot_ = −28.8 kJ/mol). The packing perspective viewed along the b-axis is illustrated in Figure 2b.

#### 2.1.2. NPhp (Nandrolone Phenylpropionate)

**Figure 3:**
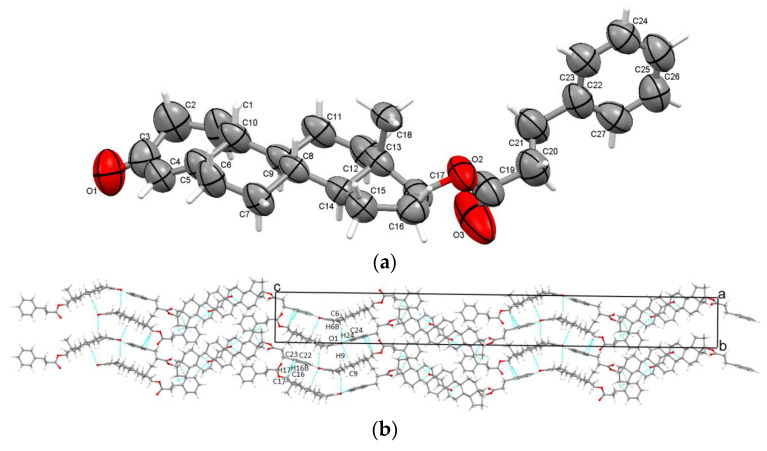
Displacement ellipsoid representations of asymmetric unit of TPhp atoms at a 50% probability level (**a**); packing along the a-axis (**b**).

The phenylpropionate ester of nandrolone crystallizes in a tetragonal system, possessing a non-centrosymmetric P4_3_2_1_2 space group with one molecule in the asymmetric unit (Figure 3a); additionally, it has unusual lattice parameters with a very long c axis (a = b = 8.0959 Å; c = 71.2184 Å). O1 carbonyl oxygen is involved in trifurcated hydrogen bonding with two neighboring C rings (C6A-H6AB···O1 with an interaction magnitude of −20.3 kJ/mol; −41.8 kJ/mol for C9-H9···O1) and −11.7 kJ/mol for C24-H24···O1 interaction with one phenyl ring. Further, the D rings are involved in the C-H···π contacts (C17-H17···C23 and C16-H16···C22) with the phenyl ring of the ester chain showing an interaction energy of −35 kJ/mol. The crystal packing perspective of NPhp, seen along the c-axis, is presented in Figure 3b.

#### 2.1.3. NCyp (Nandrolone Cypionate)

**Figure 4:**
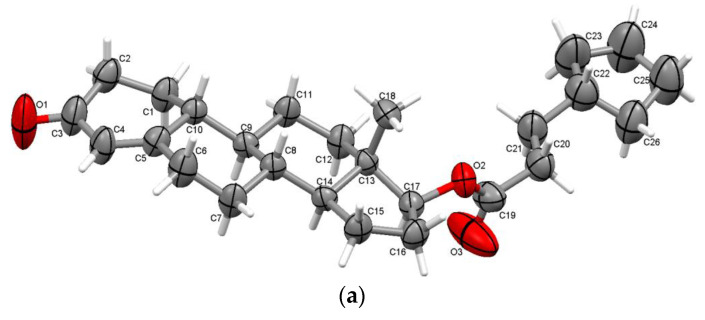

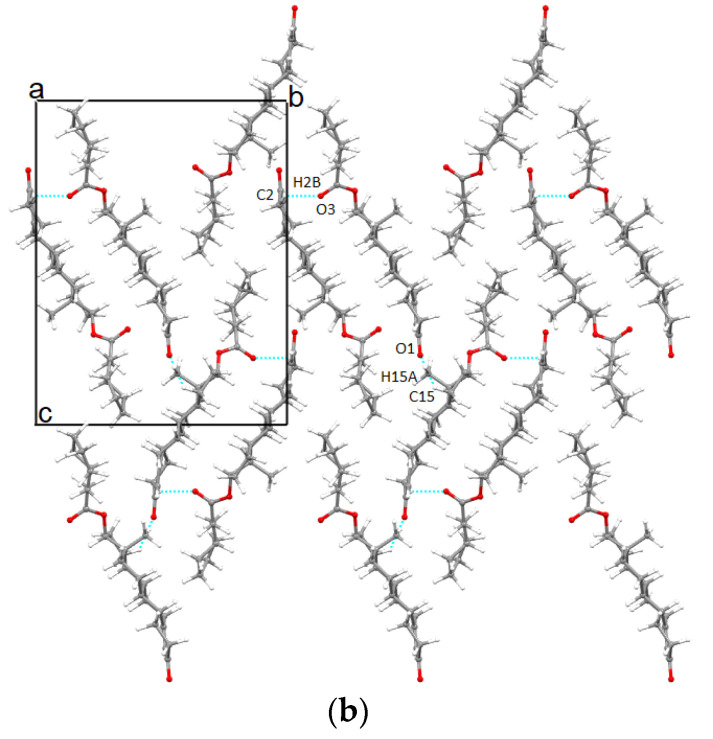
Displacement ellipsoid representations of asymmetric unit of NCyp atoms at a 50% probability level (**a**); packing along c-axis (**b**).

Cypionate ester of nandrolone is crystallizing non-centrosymmetrically in the orthorhombic P2_1_2_1_2_1_ space group and is characterized by one molecule in the asymmetric unit (Figure 4a). In the formation of supramolecular self-assemblies, combinations of C-H···O hydrogen bonds with O1 carbonyl oxygen are involved, building the C15-H15A···O1 interaction which neighbors a D steroid ring (with an interaction energy of −21.0 kJ/mol) and the carbonyl O3 oxygen bounded to the A steroid ring via a C2-H2B···O3 interaction (with a high value of interaction energy of −48.4 kJ/mol). Supramolecular arrangements packed along the a-axis are presented in Figure 4b.

#### 2.1.4. NUnd (Nandrolone Undecanoate)

**Figure 5:**
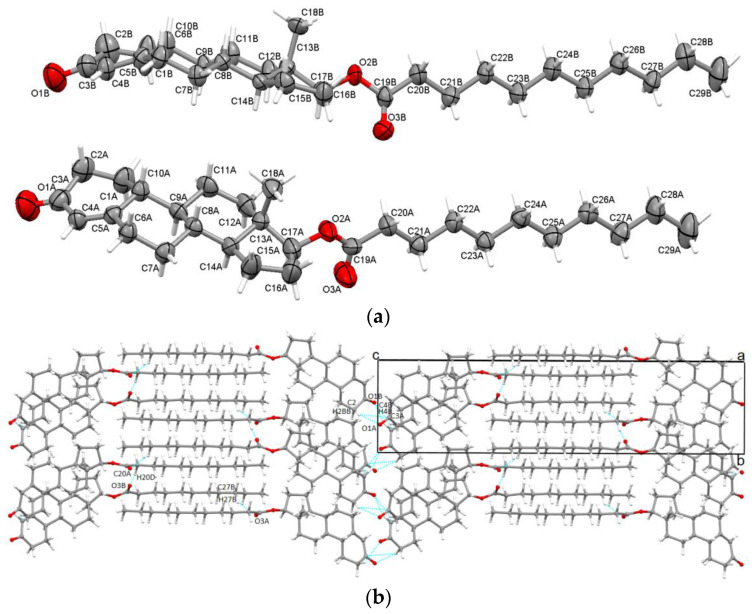
Displacement ellipsoid representations of asymmetric unit of NUnd atoms at a 50% probability level (**a**); packing along the a-axis (**b**).

Undecanoate ester is one of the longest esters available and the longest nandrolone ester investigated in the actual paper. From a crystallographic perspective, it was found to mono-clinically crystallize in the non-centrosymmetric P2_1_ space group with two steroid molecules in asymmetric unit (Figure 5a) linked via C20A-H20D···O3B and C2A-H2AA···C4B contacts with an energy of −48.2 kJ/mol. The high energy of the interaction between the two molecules in the asymmetric unit is explained by the fact that these two are almost parallel; in this way, the distances between the atoms are small. Among the esters discussed, undecanoate is the only one that has two molecules in the asymmetric unit (denoted as A and B).

Both O1A and O1B carbonyl oxygen are acceptors in bifurcated hydrogen bonds: C4A-H4A···O1A interaction with an interaction energy of −28.3 kJ/mol links two A molecules; C2B-H2BB···O1A interaction binds a neighboring B molecule (E = −34.1 kJ/mol). Further, the B molecule is linked to one A molecule via the C2A-H2AA···O1B interaction (E = −34.1 kJ/mol) and two B molecules via the C4B-H4B···O1B (E = −23.3 kJ/mol). 

Carbonyl O3A oxygen of the ester chain forms bifurcated hydrogen bonds as well and links two B steroid molecules (C20B-H20B···O3A with an energy of −54.1 kJ/mol and C27B-H27B···O3A with −34.4 kJ/mol).

Like in the previous esters, the C4=C5 double bond is involved in the C-H···π interactions, which link one A and one B molecule (C2B-H2BB···C3A contact with an interaction energy of −33.6 kJ/mol). The crystal packing diagram along the a-axis is illustrated in Figure 5b.

Through crystal structure analysis, a few structural features can be pointed out, as follows:

(i) All esters crystallized in a wide variety of non-centrosymmetric space groups of trigonal, tetragonal, orthorhombic and monoclinic crystal systems.
(ii) Asymmetric units consist of single steroid molecules in short esters (propionate and phenylpropionate) and medium (cypionate) ester and two individual molecules in the long undecanoate ester.
(iii) Despite the fact that combinations of C-H···O interactions are involved in the arrangement of supramolecular assemblies, their contributions compared to the dispersion effects is small (see Table 2 for crystal lattice energies and Table S4, Supplementary Materials, for pairwise intermolecular energies).
(iv) Hydrogen···carbonyl distances implied in C-H···O hydrogen bonding revealed similar values to those found in other analogues from the steroid group [18,19,20,21].
(v) From a conformational standpoint of steroid rings, the six-membered A rings are found to have an intermediate sofa–half-chair geometry; both the B and C rings were found to have a chair geometry; the five-membered D rings were found to have an envelope geometry. The anhydrous form of nandrolone depicts similar configurations as well [12,13].

### 2.2. Crystal Lattice Energies Analysis

Total lattice energies along with their breakdown in four distinct components were computed by the use of the atom–atom Coulomb–London–Pauli (CLP) model (Table 2). 

NPro is the shortest investigated ester and shows an energy of −57.7 kJ/mol, while the longest ester (NUnd with eleven carbons ester length) shows a value of −195.0 kJ/mol. It is observed that, through incorporating water into the NPro crystal, the lattice is destabilized, which leads to a low absolute value of 57.7 kJ/mol of the total energy. A similar behavior was also reported in the case of other crystals based on active pharmaceutical ingredients where the incorporation of solvents led to a lattice with a lower energy in absolute value [22,23,24,25,26].

In all four esters, the dispersion energies are dominant; these increase once with the increase in the number of carbon atoms in the ester. Similar results with dispersion effects playing the major role in stabilization were reported for other esterified forms of various steroids [27,28,29].

At the same time, the weight associated with the dispersion is lower in NPro compared to the rest of the esters where the dispersion has a weight of over 90% of the total energy. This can be attributed to the inclusion of water molecules in the NPro network, which increases the significance of the Coulombic term through the classical O-H···O hydrogen bonds between the steroid and water. It is clearly observed that, in the NPro crystal, the Coulombic term (E_coul_) plays a more important role than in the rest of the esters.

The polarization and repulsion energies do not show a certain trend; they are relatively constant in weight with respect the total energy in all four crystals.

### 2.3. Pairwise Intermolecular Energies Computation

The evaluation of the nature and magnitudes of the intermolecular interaction energies for the contacts characterized by shorter distances than the sum of van der Waals radii offers in-depth, qualitative and quantitative insights upon crystal packing (Table S4, Supplementary Materials). This process involves the computation of a total interaction energy which can be further divided in three attraction terms (electrostatic—E_ele_; polarization—E_pol_; dispersion—E_disp_) and one repulsive (E_rep_) term. 

Based on the energy values listed in Table S4, a few conclusions can be pointed out:

(i) Overall, the dispersion energy plays the most significant role in solid-state cohesion (similar outcome was found in Section 2.2 Crystal lattice energies analysis), followed by the electrostatic component. The polarization component has the smallest impact.
(ii) The polarization terms are the least significant in terms of adhesion, which suggests that the molecules are not polarized.
(iii) Since NPro contains water molecules, compared to NPhp, NCyp and NUnd, their presence increases the weight of the electrostatic component to the total energy of interactions through O-H···O hydrogen bonds.
(iv) Interactions taking place between molecules located roughly parallel are characterized by high values of dispersion energy as a consequence of a large number of contact atoms with a small distance between them; a good example of this is the interaction between the two NUnd molecules in the asymmetric unit.
(v) For molecular pairs located end to end, the electrostatic component becomes more significant.
(vi) Magnitudes of total interaction energies (E_tot_) were found to be low–medium and had a wide range (from −9.0 to −54.2 kJ/mol) due to the random orientations of neighboring molecules, relative to one another and in the absence of strong hydrogen bonds.

### 2.4. Hirshfeld Surfaces and Fingerprint Plots Analysis

Analysis using 3D Hirshfeld surfaces and their related 2D fingerprint plots is a useful tool that can be applied to visualize intermolecular interactions. Surfaces were generated for the asymmetric units of all four crystals. NUnd is characterized by two steroid molecules in the asymmetric unit; thus, they were treated separately. 

All intermolecular interactions which are characterized by distances shorter than the sum of van der Waals radii are presented in Table S1 (Supplementary Materials) and illustrated with arrows on the Hirshfeld surfaces (Figure S2 Supplementary Materials). The surfaces are understood and commented on based using a color coding system (red, white, blue), as follows: red areas represent strong intermolecular interactions with distances shorter than the sum of the vdW radii; white areas represent intermolecular contacts with distances which are roughly equal to the sum of the vdW radii; blue represents weak interactions with contacts longer than the sum of the vdW radii. There is an associated 2D fingerprint plot for each Hirshfeld surface (Figure S3, Supplementary Materials).

The conclusions that can be summarized following review of the Hirshfeld surfaces, the fingerprint plots and the pairs of (d_e_ and d_i_) distances (where d_i_ is the distance from an interior nucleus to the surface and d_e_ is the distance from the surface to an exterior nucleus) are as follows:

(i) Fingerprint diagrams of NPhp and NCyp (Figure S3, Supplementary Materials) are symmetrical. This is indicative of crystals with one molecule in the asymmetric unit; NPro (which is a multicomponent structure) and NUnd (characterized by two molecules in asymmetric unit) shows asymmetry in fingerprint plots due to different crystal packing environments in the solid.
(ii) The diagrams of NPro, NPhp and NUnd are characterized by protruding H···O/O···H spikes which suggests the presence of C-H···O hydrogen bonds; NCyp is lacking H···O/O···H spikes due to long donor–acceptor distances (close to the sum of vdW radii) for C-H···O hydrogen bonds.
(iii) The breakdown in quantitative contributions of fingerprint plots (Table S2, Supplementary Materials) exhibit similarities in all four crystals: high percentage for H···H contacts, medium contribution for O···H/H···O contacts and a small percentage for C···H/H···C. These suggest that hydrogen bonds and weak van der Waals interactions ensure stability.
(iv) The percentage of H···O/O···H contacts in NPro is slightly higher compared to the others due to the water molecules which build O-H···O interactions and increase the weight of the Coulombic effects in the overall crystal stability.
(v) High percentages for H···H contacts (breakdown of fingerprint plots in Table S4, Supplementary Materials) supported by lattice and intermolecular interaction energies (Tables S2 and S3 Supplementary Materials) led us to conclude that dispersion effects govern the crystal packing.

### 2.5. Evaluation of Ester Solubility

**Figure 6:**
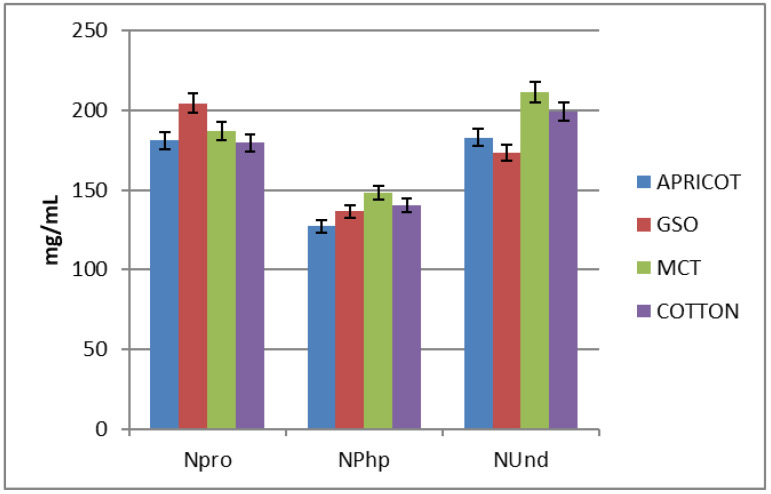
Graphical diagram of esters’ solubility. Abbreviations: GSO—grape seed oil; MCT—medium-chain triglyceride.

The solubility of esters are graphically illustrated in Figure 6 and their values listed in Table S3 (Supplementary Materials). For cypionate ester most likely the evaluation was not successful due to the fact that it shows a clay appearance and has a low melting point at room temperature, causing the solutions to become gelatinous and transparent mixtures. The values of the other three esters do not show any correlation between the length of the ester chain and solubility. The shortest propionate ester (NPro) possesses a solubility that averages roughly 188.1 mg/mL. The medium phenylpropionate ester (NPhp) shows a lower solubility compared to the other two, at approximately 138 mg/mL. The longest undecanoate ester (NUnd) has a solubility of about 191.7 mg/mL, which is comparable to that of the propionate.

Similar solubilities were reported for the analogue esters of testosterone: 175.5 mg/mL for testosterone propionate, 139 mg/mL for testosterone phenylpropionate [30], 255 mg/mL for testosterone cypionate and 197 mg/mL for the undecanoate ester of testosterone [31].

Two polymorphs of hydroxyprogesterone caproate have been reported to possess solubilities of 278 mg/mL and 301 mg/mL, respectively, at an ambient temperature of 20 °C in mixtures of castor oil [32].

### 2.6. FT-IR Spectroscopy Analysis

As was shown using single-crystal X-Ray diffraction, NPro is found in a hydrate form. The FTIR diagram of NPro is characterized by absorption bands which appear at high frequencies (3512, 3459 and 3288 cm^−1^) due to O-H stretching in water molecules and hydrogen bonds between functional groups from NPro (see Section 2.1.1). For NCyp (3444 cm^−1^) and, to a smaller degree, for NUnd, broad bands are shown, which are assigned to O-H stretching of surface water; neither of these esters contain water molecules of recrystallization. 

The absorption bands that appear in the 2800–3060 cm^−1^ wavenumbers (Figure S4 Supplementary Materials) are similar to each other and can be assigned to symmetric and asymmetric C-H stretching of the CH, CH_2_ and CH_3_ functional groups.

Two absorption bands appearing between 1649–1740 cm^−1^ wavenumbers are assigned to the carbonyl C=O groups of carbonyl (at the A rings) and ester chains. At lower frequencies (1618–1621 cm^−1^), less intense peaks are observed and attributed to C=C stretching of the carbons found in the steroid A rings. For small wave numbers (fingerprint region below 1500 cm^−1^), the spectra of NPro and NUnd are similar because the difference between them consists of a different number in the two carbon chains: NPro has three carbons and NUnd has eleven. Also, the spectra of NPhp and NCyp are similar because the differences between the two structures are given only by the phenyl ring for NPhp and cyclopentyl for NCyp.

### 2.7. DTA/TG Analysis

DTA/TG diagrams of analyzed esters are presented in Figure S5 (Supplementary Materials). Endothermic peaks are present at 78 °C for NPro, 102 °C for NPhp, 33 and 66 °C in two steps for NCyp and 66 °C for NUnd; these indicate the melting points of the compounds. Note that, for NCyp, its texture shows a clay-like appearance and possesses the lowest melting point even at ambient temperatures; during the evaluation of solubility, the solutions became gelatinous. As shown using X-Ray diffraction, there were water molecules embedded in the crystal lattice of the propionate ester. The thermogravimetric curve shows a mass loss with the onset at roughly 45 °C up to 119 °C, which is assigned to the loss of water. The exothermic phenomenon at 261 °C for NPro indicates an oxidation of the sample, which is immediately followed by decomposition.

Further endothermic peaks appeared with temperature increases: 314 °C for NPro, 370 °C for NPhp, 365 °C for NCyp and in two steps for NUnd (262 and 369 °C). These effects are attributed to samples degradation and were accompanied by a noticeable mass loss.

Further wide exothermic events (roughly around 450 °C) are attributable to decomposition and oxidation of the remaining samples accompanied with a small mass loss among the remaining materials. A similar behavior was highlighted for other pharmaceutical compounds, such as riboflavin and norfloxacin [33].

## 3. Materials and Methods

### 3.1. Materials and Recrystallization Experiments

At room temperature, the materials are yellow crystalline powders (NPro and NUnd), a white powder (NPhp) and an orange gel mixture (NCyp), requiring different recrystallization methods. Single crystals that were suitable for X-ray data analyses were grown in various alcohols: methanol (NPro, NPhp), propanol (NUnd) and butanol (NCyp).

### 3.2. Powder X-ray Diffraction

The powder diffraction patterns of esters were recorded with a Bruker D8 Advance diffractometer with the X-Ray tube operating at 40 kV and 40 mA, equipped with a germanium monochromator used in order to filter only the CuKα1 radiation and a LINXEYE detector. X-ray diffraction patterns for samples were scanned at a range of 2θ = 3–40° using the DIFFRAC plus XRD Commander program with a scanning speed of 0.01°/s.

### 3.3. Single-crystal X-ray Diffraction and Structures Refinement

Diffraction intensities were collected with a SuperNova diffractometer with dual micro-source (Cu and Mo) and the X-ray tube set at 50 kV and 0.8 mA. Collection of data, reduction, Lorentz, polarization and absorption correction was accomplished using CrysAlis PRO software (Version 40_64.84a) [34]. The structures of NPro, NCyp and NUnd were determined using SHELXT (https://scripts.iucr.org/cgi-bin/paper?S2053273314026370, accessed on 15 October 2023) [35]; the structures of NPhP were determined using SHELXS [36]. They were further refined using least squares minimization in SHELXL [37]. Al methods are part of the Olex2 software (Version 1.2.10) [38]. 

A riding model was applied for hydrogen atoms refinement with the isotropic displacement parameter U_iso_(H) = 1.2U_eq_(C) for ternary CH groups [C-H = 0.93 Å] and secondary CH_2_ groups [C-H = 0.97 Å], and 1.5U_eq_(C) for CH_3_ methyl groups [C-H = 0.96 Å].

### 3.4. Computational Programs

During computation, the C-H and O-H bond distances for all OH, CH, CH_2_ and CH_3_ functional groups were moved to the normalized distances C-H = 1.083 Å and O-H = 0.993 Å. Computational procedures were performed using the fractional coordinates of atoms in the unit cells (CIF files) obtained by single-crystal X-ray diffraction. 

In the CLP-Pixel software (http://www.angelogavezzotti.it/, accessed on 15 October 2023) [39], the Coulomb–London–Pauli approach was utilized to calculate the crystal energies. This approach involves the evaluation of three attractive terms, which are the Coulombic energy, the polarization energy and the dispersion energy, as well as one repulsive term. Through a consideration of these energies, the software computes the total lattice energies of the crystals under study.

The intermolecular interaction energies can be divided into four categories, namely electrostatic, polarization, dispersion and exchange repulsion [40]. Pairs of molecules with contact distances shorter than the sum of van der Waals radii were computed using CrystalExplorer software (Version 21.5) [41]. The B3LYP/6-31G(d,p) wave function at the level of theory was used considering the following scale factors: k_ele_ = 1.057, k_pol_ = 0.740, k_disp_ = 0.871, k_rep_ = 0.618.

The Hirshfeld surfaces and fingerprint plots for each crystal were generated using the d_norm_ function in the CrystalExplorer software [41].

### 3.5. Solubility Evaluation

From a pharmaceutical standpoint, the solubility of esterified agents (mg/mL) plays an important role in drug development and delivery. In this context, the solubilities were evaluated in multiple mixtures based on various organic oils: medium-chain triglyceride (MCT), apricot, grape seed oil (GSO) and cottonseed oil. Each solution contains a mixture of benzyl alcohol, benzyl benzoate and oil with a volumetric ratio of 2% benzyl alcohol, 20% benzyl benzoate and the rest of 78% oil.

Pharmaceuticals preparations of different lipophilic drugs (esterified forms of steroids being included) use benzyl benzoate as the main co-solvent (solubilizer); benzyl alcohol prevents the microbial development and growth and possesses solvent properties; the oils are carriers and, to a smaller degree, the solvents are as well.

Solubility evaluation was accomplished at a room temperature of 24 °C in successive additions of small quantities of raw materials (a few mg each step). The mixtures were stirred for up to several hours in order to achieve a completely dissolved solution. In the case that an undissolved excess of raw materials remained in the suspension, small quantities of mixtures were added until the solutions became perfectly homogenous and clear. Two measurements were performed in order to attain a good accuracy; their average was further discussed.

### 3.6. FT-IR Spectroscopy

To obtain the FT-IR spectra of the analyzed samples, the Jasco 6200 spectrometer (JASCO Corporation, Tokyo, Japan) was used, operating with 256 scans and with a resolution of 4 cm^−1^ in a spectral range of 4000–400 cm^−1^. The samples were prepared in the form of pellets based on KBr.

### 3.7. Differential Thermal Analysis (DTA) and Thermogravimetric Analysis (TG)

DTA/TGA curves were recorded using a Schimadzu DTG-60 analyzer (SHIMADTZU CORPORATION, Kyoto, Japan) that simultaneously measures DTA and TGA curves. An alumina sample cell with the dimensions of 5.8 × 2.5 mm^2^ was used, the heating speed of the samples being 10 °C/min under the purging action with dry nitrogen having a flow rate of 70 mL/min.

## 4. Conclusions

The crystal structures of some nandrolone-based esters, including propionate, phenylpropionate, cypionate and undecanoate, were determined and reported. They crystallize in various rare non-centrosymmetric space groups: trigonal P3_1_21 for propionate and tetragonal P4_3_2_1_2 for phenylpropionate. Meanwhile, orthorhombic P2_1_2_1_2_1_ forms for cypionate and a monoclinic P2_1_ space group forms for undecanoate ester. Backbone steroid rings exhibit a sofa–half-chair conformation in the A rings, a chair conformation in the six-membered B and C rings, and an envelope conformation in the five-membered D rings. The results obtained from the computational methods indicate that supramolecular architectures are primarily stabilized through dispersion effects. The interactions involving C-H···O hydrogen bonding to carbonyl groups, while present, play a smaller role in determining the overall crystal stability and packing.

The solubility evaluation shows that various added ester functionalities led to comparable values (but slightly lower in the phenylpropionate form) which do not depend on the length of the ester chain.

## Figures and Tables

**Table 1:** Crystal structures and refinement data details.

Identification Code	NPro (Nandrolone Propionate)	NPhp (Nandrolone Phenylpropionate)	NCyp (Nandrolone Cypionate)	NUnd (Nandrolone Undecanoate)
Empirical formula	C_21_H_31_O_3.5_	C_27_H_34_O_3_	C_26_H_38_O_3_	C_29_H_46_O_3_
Formula weight	335.46	404.52	398.56	442.66
Temperature/K	293(2)	293(2)	293(2)	293(2)
Crystal system	trigonal	tetragonal	orthorhombic	monoclinic
Space group	P3_1_21	P4_3_2_1_2	P2_1_2_1_2_1_	P2_1_
a/Å	7.7610(2)	8.09590(10)	7.64320(10)	9.9889(6)
b/Å	7.7610(2)	8.09590(10)	15.3112(4)	8.2547(5)
c/Å	56.2110(18)	71.2184(14)	19.7800(4)	32.9847(14)
α/°	90	90	90	90
β/°	90	90	90	94.761(5)
γ/°	120	90	90	90
Volume/Å^3^	2932.16(18)	4667.91(15)	2314.79(8)	2710.4(3)
Z	3	8	4	4
ρ_calc_g/cm^3^	1.152	1.151	1.144	1.085
μ/mm^−1^	0.593	0.576	0.566	0.068
F(000)	1098.0	1744.0	872.0	976.0
Crystal size/mm^3^	0.05 × 0.04 × 0.03	0.09 × 0.04 × 0.01	0.08 × 0.01× 0.01	0.08 × 0.06 × 0.01
Radiation	CuKα (λ = 1.54184)	CuKα (λ = 1.54184)	CuKα (λ = 1.54184)	MoKα (λ = 0.71073)
2Θ range for data collection/°	9.44 to 146.39	9.93 to 141.28	7.30 to 141.89	5.75 to 57.55
Index ranges	−9 ≤ h ≤ 9, −9 ≤ k ≤ 9, −68 ≤ l ≤ 68	−9 ≤ h ≤ 9, −9 ≤ k ≤ 8, −86 ≤ l ≤ 85	−9 ≤ h ≤ 9, −18 ≤ k ≤ 18, −23 ≤ l ≤ 20	−10 ≤ h ≤ 13, −11 ≤ k ≤ 10, −43 ≤ l ≤ 41
Reflections collected	41084	16752	34086	22788
Independent reflections	3777 [R_int_ = 0.0614, R_sigma_ = 0.0216]	4401 [R_int_ = 0.0214, R_sigma_ = 0.0220]	4429 [R_int_ = 0.0297, R_sigma_ = 0.0138]	11283 [R_int_ = 0.0856, R_sigma_ = 0.1698]
Data/restraints/parameters	3777/0/227	4401/0/272	4429/0/263	11283/1/581
Goodness-of-fit on F^2^	1.045	1.023	1.061	0.978
Final R indexes [I ≥ 2σ (I)]	R_1_ = 0.0680, wR_2_ = 0.2032	R_1_ = 0.0694, wR_2_ = 0.2045	R_1_ = 0.0652, wR_2_ = 0.1986	R_1_ = 0.0865, wR_2_ = 0.1462
Final R indexes [all data]	R_1_ = 0.0827, wR_2_ = 0.2210	R_1_ = 0.1089, wR_2_ = 0.2338	R_1_ = 0.0710, wR_2_ = 0.2089	R_1_ = 0.2801, wR_2_ = 0.2189
Largest diff. peak/hole/e Å^−3^	0.55/−0.18	0.25/−0.18	0.36/−0.23	0.23/−0.14
Flack parameter	0.11(15)	0.03(12)	0.04(8)	0.2(10)

**Table 2:** Crystal lattice energies.

Structure	Molar Mass g/mol	E_coul_ (kJ/mol)	E_pol_ (kJ/mol)	E_disp_ (kJ/mol)	E_rep_ (kJ/mol)	E_latt_ (kJ/mol)
NPro	338.95	−15.1	−17.0	−43.1	17.7	−57.5
NPhp	404.52	−20.9	−44.8	−156.5	52.3	−169.9
NCyp	398.56	−9.1	−56.3	−159.9	59.1	−166.2
NUnd	442.66	−19.9	−64.0	−181.1	70.7	−195.0

E_coul_: Coulombic energy; E_pol_: polarization energy; E_disp_: dispersion energy; E_rep_: repulsion energy; E_latt_: total crystal lattice energy.

## Data Availability

CIF files of analyzed nandrolone esters were deposited with the Cambridge Crystallographic Data Centre with the following deposition numbers: 2285512 (NPro); 2285513 (NPhp); 2285514 (NCyp); 2285515 (NUnd). Copies of the CIF files can be obtained free of charge on written application to CCDC, 12 Union Road, Cambridge, CB2 1EZ, UK (Fax: +44-1223-336033); on request via e-mail to deposit@ccdc.cam.uk or by access to http://www.ccdc.cam.ac.uk (accessed on 15 October 2023).

## Supplementary Materials

The following supporting information can be downloaded at: https://www.mdpi.com/article/10.3390/molecules28207179/s1, Figure S1: Powder X-Ray diffraction patterns comparison experimental (Exp) and simulated (Sim): NPro (a), NPhp (b), NCyp (c), NUnd (d); Figure S2: Hirshfeld surfaces mapped with d_norm_ ilustrating the contacts referred in Table S1. Surfaces were represented with the clour scale in the ranges as follows: NPro (a) −0.51 (red) to 1.52 (blue), −0.10 (red) to 1.69 (blue) for NPhp, −0.09 (red) to 1.68 (blue) for NCyp, 0.01 (white) to 1.55 (blue) for NUnd; Figure S3: Fingerprint plots of analysed nandrolone esters; Figure S4: FT-IR spectra of analyzed nandrolone esters; Figure S5: Thermal DTA/TG diagrams of analyzed esters; Table S1: Intermolecular interactions for studied crystals (Å, ^o^); Table S2: Contributions to the Hirshfeld surfaces for various intercontacts; Table S3: Solubility of esters in various mixtures; Table S4: Nature and magnitudes of intermolecular interaction energies for selected contacts (kJ/mol). CIF files of analyzed nandrolone esters were deposited with the Cambridge Crystallographic Data Centre with the following deposition numbers: 2285512 (NPro); 2285513 (NPhp); 2285514 (NCyp); 2285515 (NUnd). Copies of the CIF files can be obtained free of charge on written application to CCDC, 12 Union Road, Cambridge, CB2 1EZ, UK (Fax: +44-1223-336033); on request via e-mail to deposit@ccdc.cam.uk or by access to http://www.ccdc.cam.ac.uk (accessed on 15 October 2023).
