# Supplementary material for: Crystal Structure and Intermolecular Energy for Some Nandrolone Esters
Source: Molecules. 2023 Oct 19;28(20):7179. doi: 10.3390/molecules28207179 (PMC10609429; doi:10.3390/molecules28207179)
Supplement: Supplementary file 1 [file molecules-28-07179-s001.zip › molecules-2592092-supplementary.pdf]

Supporting information

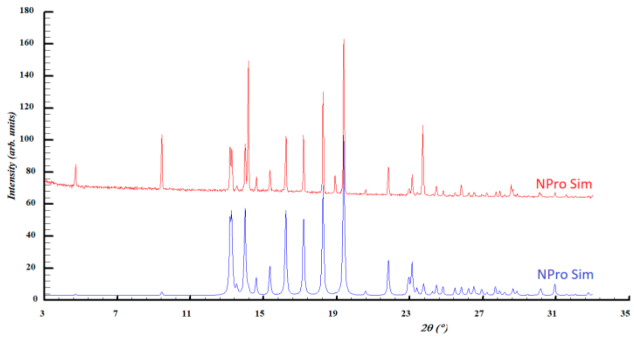

(a)

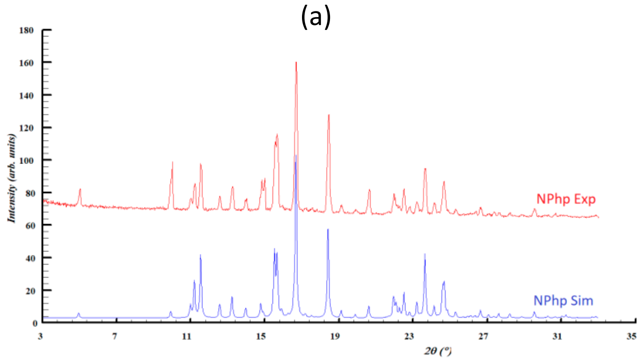

(b)

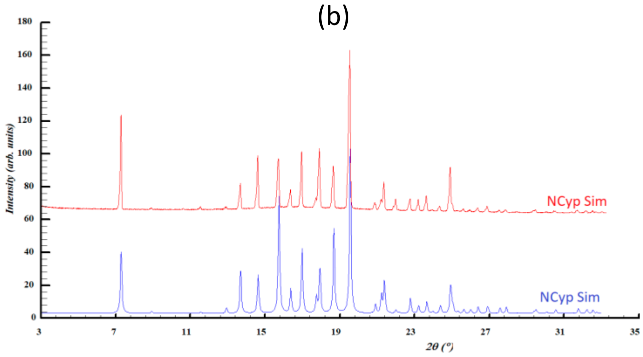

(c)

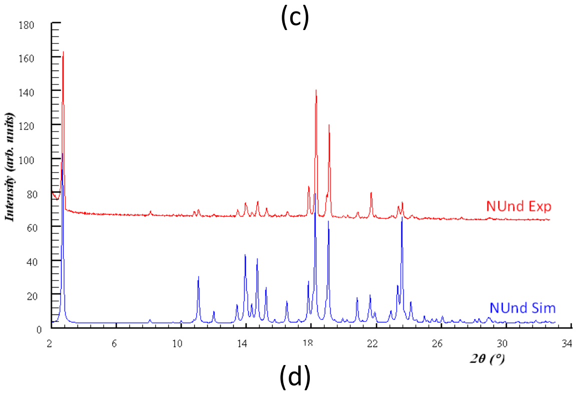

(d)

Figure S1. Powder X-Ray diffraction patterns comparison experimental (Exp) and simulated (Sim): NPro (a), NPhp (b), NCyp (c), NUnd (d)

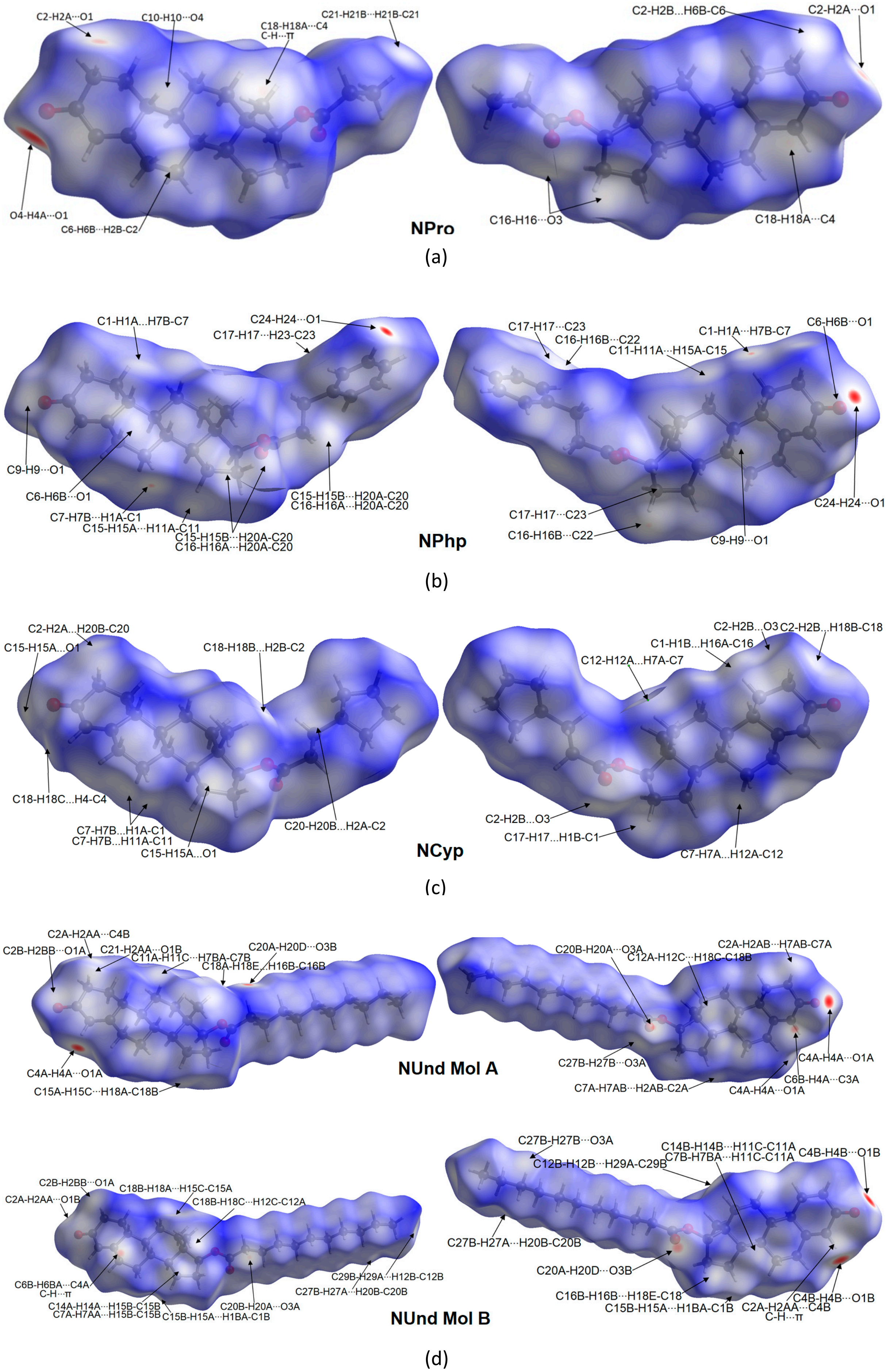

C2-H2A···O1
C10-H10···O4
C18-H18A···C4
C-H···π
C21-H21B···H21B-C21
O4-H4A···O1
C6-H6B···H2B-C2
NPro
C2-H2B···H6B-C6
C2-H2A···O1
C16-H16···O3
C18-H18A···C4
(a)
C1-H1A...H7B-C7
C24-H24···O1
C17-H17···H23-C23
C9-H9···O1
C6-H6B···O1
C7-H7B···H1A-C1
C15-H15A···H11A-C11
C15-H15B···H20A-C20
C16-H16A···H20A-C20
NPhp
C17-H17···C23
C16-H16B···C22
C1-H1A···H7B-C7
C11-H11A···H15A-C15
C6-H6B···O1
C24-H24···O1
C9-H9···O1
(b)
C2-H2A...H20B-C20
C15-H15A...O1
C18-H18B...H2B-C2
C18-H18C...H4-C4
C7-H7B...H1A-C1
C7-H7B...H11A-C11
C15-H15A...O1
C20-H20B...H2A-C2
NCyp
C2-H2B...O3
C2-H2B...H18B-C18
C1-H1B...H16A-C16
C12-H12A...H7A-C7
C17-H17...H1B-C1
C7-H7A...H12A-C12
(c)
C2A-H2AA···C4B
C2B-H2BB···O1A
C21-H2AA···O1B
C11A-H11C···H7BA-C7B
C20A-H20D···O3B
C18A-H18E...H16B-C16B
C4A-H4A···O1A
C15A-H15C···H18A-C18B
NUnd Mol A
C20B-H20A···O3A
C12A-H12C···H18C-C18B
C2A-H2AB···H7AB-C7A
C27B-H27B···O3A
C4A-H4A···O1A
C6B-H4A···C3A
C7A-H7AB···H2AB-C2A
C2B-H2BB···O1A
C2A-H2AA···O1B
C18B-H18A···H15C-C15A
C18B-H18C···H12C-C12A
C6B-H6BA···C4A
C-H···π
C14A-H14A···H15B-C15B
C7A-H7AA···H15B-C15B
C15B-H15A···H1BA-C1B
C20B-H20A···O3A
C29B-H29A···H12B-C12B
C27B-H27A···H20B-C20B
NUnd Mol B
C27B-H27B···O3A
C12B-H12B···H29A-C29B
C14B-H14B···H11C-C11A
C7B-H7BA···H11C-C11A
C4B-H4B···O1B
C27B-H27A···H20B-C20B
C20A-H20D···O3B
C16B-H16B···H18E-C18
C15B-H15A···H1BA-C1B
C2A-H2AA···C4B
C-H···π
C4B-H4B···O1B
(d)

Figure S2. Hirshfeld surfaces mapped with d_{norm} illustrating the contacts referred in Table S1. Surfaces were represented with the clour scale in the ranges as follows: NPro (a) -0.51 (red) to 1.52 (blue), -0.10 (red) to 1.69 (blue) for NPhp, -0.09 (red) to 1.68 (blue) for NCyp, 0.01 (white) to 1.55 (blue) for NUnd.

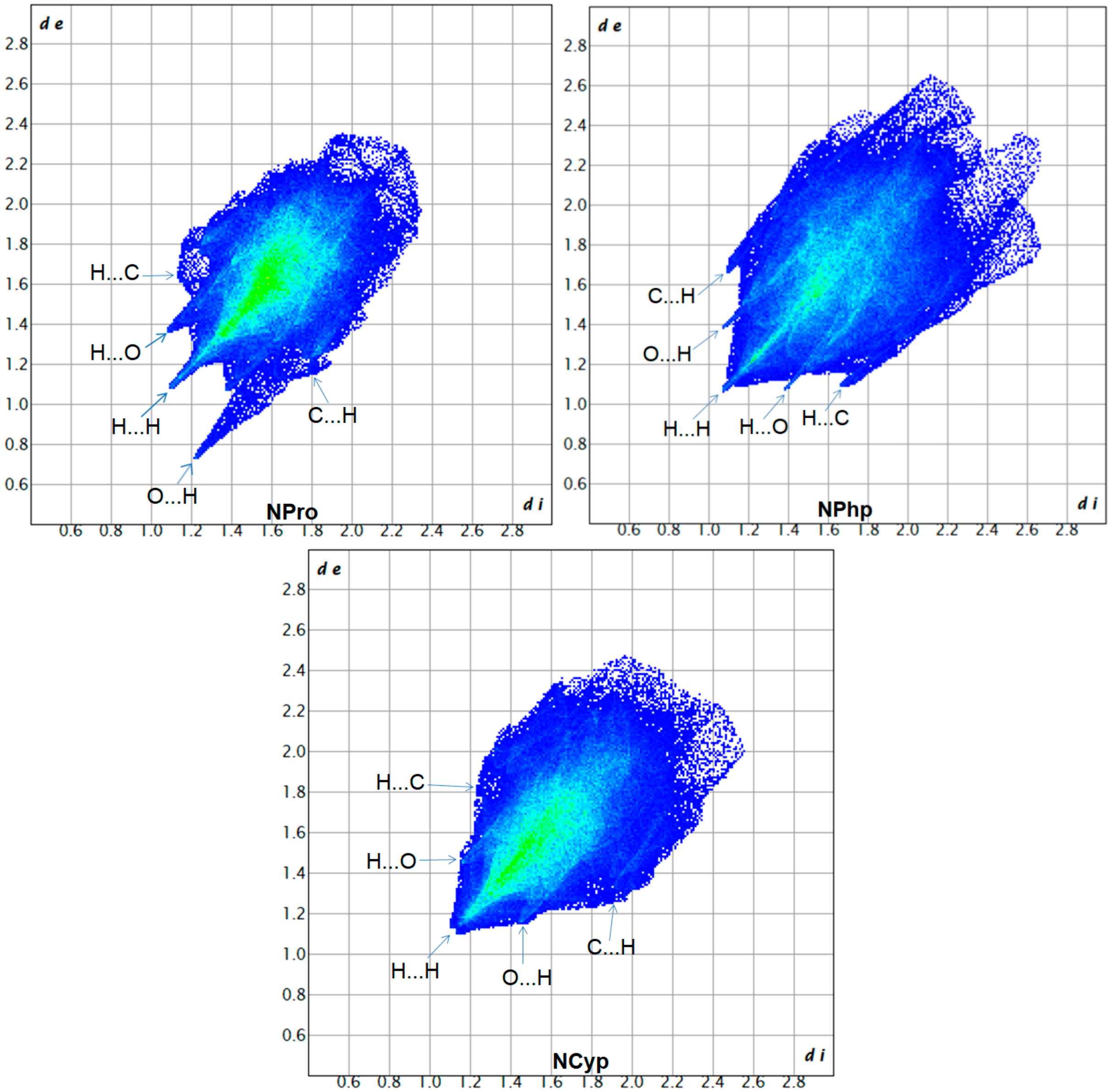

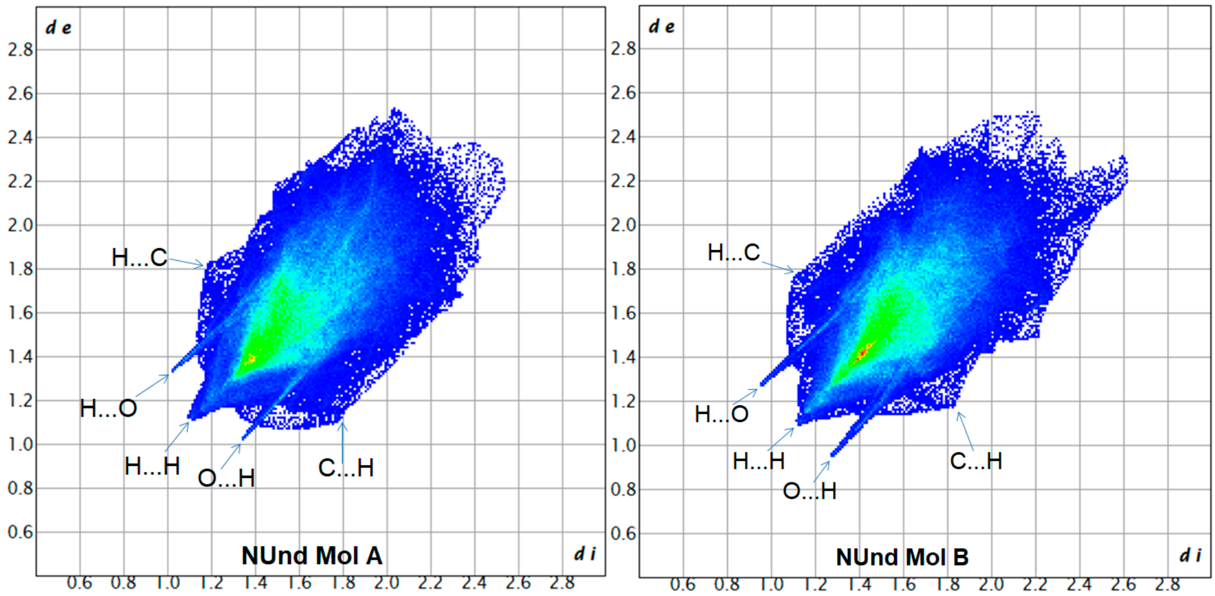

Figure S3. Fingerprint plots of analysed nandrolone esters

Table S1. *Intermolecular interactions for studied crystals (Å, °)*

**Structure**	**D-H···A**	**D-H**	**H···A**	**D···A**	**<(D-H···A)**
NPro	O4-H4A···O1	0.993	1.951(1)	2.829(3)	145.9(2
	C2-H2A···O1	1.089	2.454(2)	3.219(1)	126.2(8)
	C18-H18A···OC4	1.089	2.771(1)	3.356(5)	113.4(5)
	C10-H10···O4	1.089	2.688(5)	3.650(1)	146.9(8)
	C16-H16···O3	1.089	2.568(3)	3.313(2)	124.8(4)
	C6-H6B···H2B-C2	1.089		2.179(2)	
	C21-H21B···H21B-C21	1.089		2.263(3)	
NPhp	C9-H9···O1	1.089	2.682(1)	3.746(2)	165.3(6)
	C24-H24···O1	1.089	2.456(3)	3.473(3)	154.8(2)
	C6-H6B···O1	1.089	2.663(3)	3.6145)	145.5(5)
	C6-H6B···H24-C24	1.089		2.344(3)	
	C16-H16A···H20A-C20	1.089		2.323(1)	
	C15-H15B···H20A-C20	1.089		2.224(5)	
	C16-H16B···C22	1.089	2.756(6)	3.769(6)	154.5(1)
	C17-H17···C23	1.089	2.864(3)	3.692(5)	132.8(8)
	C15-H15A···H11A-C11	1.089		2.312(3)	
	C1-H1A···H7B-C7	1.089		2.134(3)	
	C1-H1A···H7B-C7	1.089			
NCyp	C15-H15A···O1	1.089	2.601(1)	3.457(2)	134.9(6)
	C2-H2B···O3	1.089	2.607(5)	3.451(2)	133.7(3)
	C4-H4···H18-C18	1.089		2.281(2)	
	C4-H4···H18-C18	1.089		2.231(3)	
	C1-H1B···H17-C17	1.089		2.371(1)	
	C1-H1B···H16A-C16	1.089		2.319(2)	
	C7-H17A···H12A-C12	1.089		2.363(2)	
	C18-H18B···H2B-C2	1.089		2.231(2)	
NUnd	C20A-H20D···O3B	1.089	2.447(4)	3.475(2)	156.8(4)
	C4A-H4A···O1A	1.089	2.358(4)	3.429(4)	167.4(5)

	C27B-H27B···O3A	1.089	2.717(3)	3.692(5)	148.8(5)
	C20B-H20A···O3A	1.089	2.546(3)	3.634(6)	176.2(6)
	C2B-H2BB···O1A	1.089	2.713(4)	3.750(3)	159.1(2)
	C4B-H4B···O1B	1.089	2.219(4)	3.249(5)	156.8(7)
	C2A-H2AA···C4B	1.089	2.836(3)	3.748(4)	141.3(5)
	C27B-H27B···O3A	1.089	2.717(5)	3.692(5)	148.8(6)
	C20B-H20A···O3A	1.089	2.546(7)	3.634(5)	176.2(6)
	C6B-H6BA···C4A	1.089	2.686(2)	3.732(3)	160.9(4)
	C11A-H11C···H14B-C14B	1.089		2.362(5)	
	C11A-H11C···H7BA-C7B	1.089		2.308(3)	
	C18A-H18E···H16B-C16B	1.089		2.213(3)	
	C15A-H15C···H18A-C18B	1.089		2.387(5)	
	C18B-H18C···H12C-C12A	1.089		2.222(2)	
	C7A-H7AB···H2AB-C2A	1.089		2.388(6)	
	C15B-H15B···H14A-C14A	1.089		2.298(5)	
	C15B-H15B···H7AA-C7A	1.089		2.304(3)	
	C29B-H29A···H12B-C12B	1.089		2.304(5)	
	C27B-H27A···H20B-C20B	1.089		2.399(5)	
	C1B-H1BA···H15A-C15B	1.089		2.297(4)	

***Table S2**. Contributions to the Hirshfeld surfaces for various intercontacts*

Structure	H···H	O···H/H···O	C···H/H···C	C···O/O···C	O···O	C···C
NPro	76.6%	18.5%	4.5%	-	-	0.3%
NPhp	71.1%	16.7%	11.8%	-	-	0.4%
NCyp	80.2%	16.6%	3.1%	0.1%	-	0.2%
NUnd Mol A	84.4%	12.6%	2.6%	0.1%	-	0.3%
NUnd Mol B	84.4%	12.8%	2.4%	0.1%	-	0.3%

***Table S3.** Solubility of esters in various mixtures*

	APRICOT (mg/mL)	GSO (mg/mL)	MCT (mg/mL)	COTTON (mg/mL)
NPro	181.1	204.5	187.1	179.7
NPhp	127.2	136.6	148.3	140.2
NUnd	183.0	173.2	211.4	199.1

***Table S4.** Nature and magnitudes of intermolecular interaction energies for selected contacts (kJ/mol)*

Crystal	Interaction pair	Selected contact	E_{ele}	E_{pol}	E_{disp}	E_{rep}	E_{tot}

**NPro**	Steroid-Steroid	C16-H16B···O3	-13.5	-4.1	-22.9	8.6	-31.9
	Steroid-Steroid	C18-H18A···C4	-5.8	-1.9	-30.8	9.7	-28.8
	Steroid-Steroid	C2-H2A···O1	-3.8	-2.5	-8.1	5.4	-9.0
	Steroid-Steroid	C6-H6B···H2B-C2	-0.6	-1.0	-21.42	6.1	-16.9
	Steroid-Water	O4-H4A···O1	-26.9	-5.3	-5.3	19.1	-18.4
**NPhp**	Steroid-Steroid	C20-H20A···H15B-C16<br>C20-H20A···H16A-C16	-10.7	-1.2	-68.2	25.9	-54.2
	Steroid-Steroid	C24-H24···O1	-5.7	-1.8	-4.5	0	-12.0
	Steroid-Steroid	C15-H15A···H11-C11<br>C7-H7B···H1A-C1	-0.2	-0.5	-20.2	7.8	-13.1
	Steroid-Steroid	C17-H17 ···C23<br>C16-H16B···C22	-8.3	-2.4	-34.8	10.2	-35.3
	Steroid-Steroid	C9-H9···O1	-12.1	-4.1	-25.6	0	-41.8
	Steroid-Steroid	C6-HAB···O1	-5.1	-4.0	-27.5	0	-36.6
**NCyp**	Steroid-Steroid	C2-H2B···O3	-14.3	-6.0	-48.7	20.6	-48.4
	Steroid-Steroid	C18-H18B···H2B-C2<br>C20-H20B···H2A-C2	-5.4	-2.7	-28.0	9.7	-26.4
	Steroid-Steroid	C15-H15A···O1<br>C4-H4···H18C-C18	-4.3	-2.5	-14.2	0	-21.0
	Steroid-Steroid	C7-H7B···H1A-C1<br>C7-H7B ···H11A-C11	-1.6	-0.5	-38.0	12.4	-27.7
**NUnd**	Molecule A-Molecule B (asym unit)	C20A-H20D···O3B<br>C2A-H2AA···C4B	-7.2	-3.2	-59.2	21.4	-48.2
	Molecule A-Molecule A	C20B-H20A···O3A<br>C6B-H6BA···C4A	-12.4	-3.1	-62.5	23.9	-54.1
	Molecule A-Molecule B	C27B-H27B···O3A	-4.3	-1.9	-28.2	0.0	-34.4
	Molecule A-Molecule B	C2A-H2AA···O1B<br>C2B-H2BB···O1A	-7.2	-2.3	-24.6	0.0	-34.1
	Molecule A-Molecule A	C4A-H4A···O1A	-19.1	-3.2	-6.0	0.0	-28.3
	Molecule B-Molecule B	C27B-H27A···H20B-C20B<br>C29B-H29A···H12B-C12B	15.4	-0.7	-54.7	0.0	-40.0
	Molecule B-Molecule B	C4B-H4B···O1B	-13.0	-3.8	-6.6	0.0	-23.3
	Molecule A-Molecule B	C1B-H1BA···H15A-C15B	0.1	-0.4	-19.2	5.4	-14.1
	Molecule A-Molecule B	C2B-H2BB···C3A	-7.2	-2.7	-23.8	0	-33.7

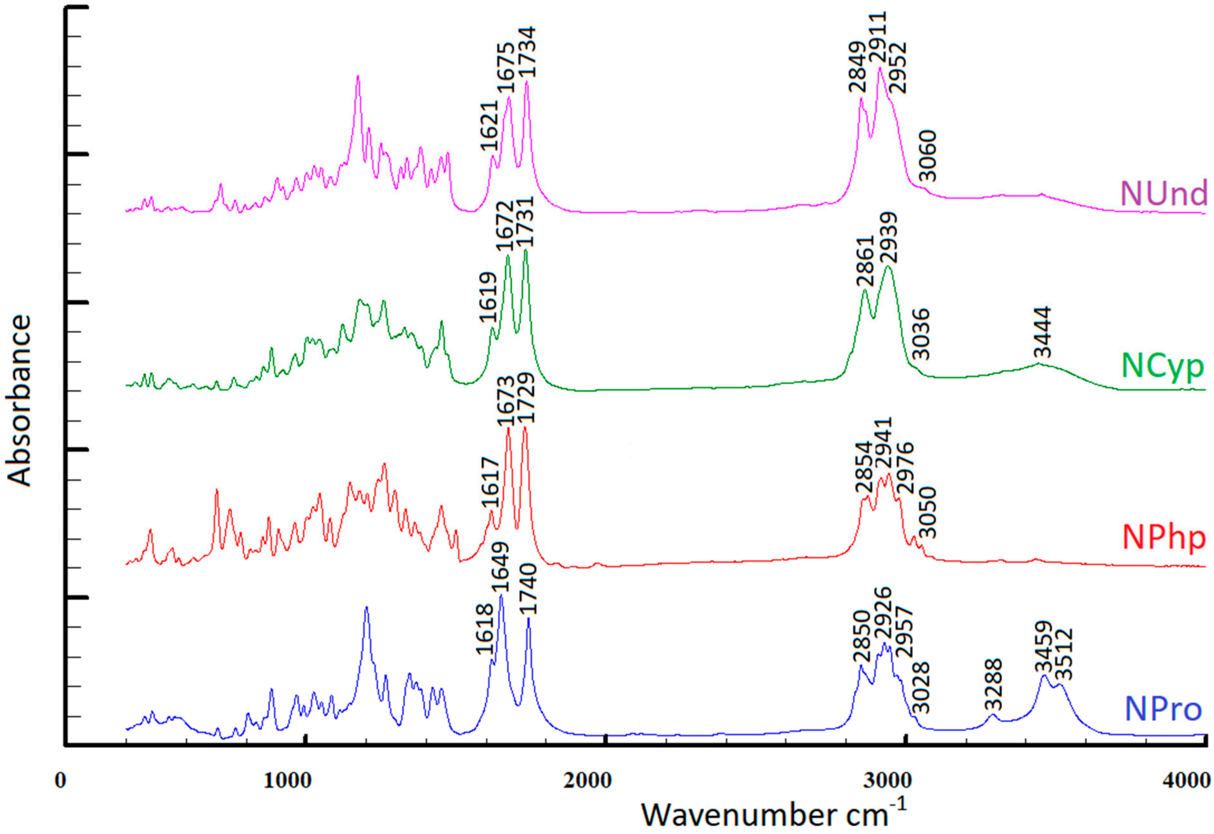

Figure S4. FT-IR spectra of analyzed nandrolone esters

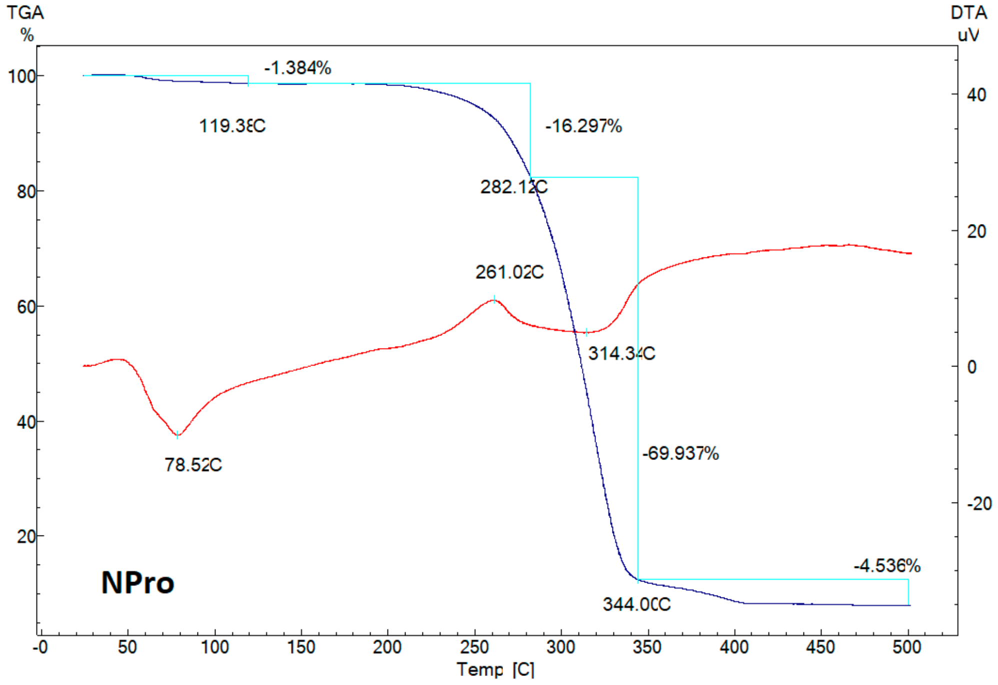

(a)

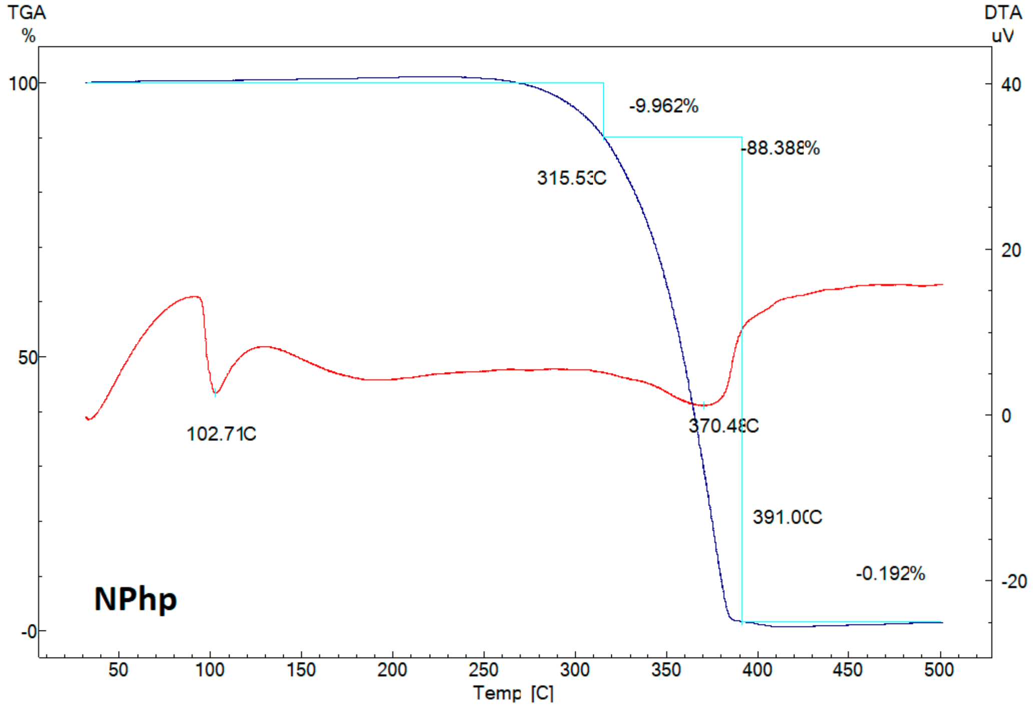
TGA
%
DTA
uV
100
50
-0
40
20
0
-20
-9.962%
-88.388%
315.53C
102.71C
370.48C
391.00C
-0.192%
NPhp
50
100
150
200
250
300
350
400
450
500
Temp [C]

(b)

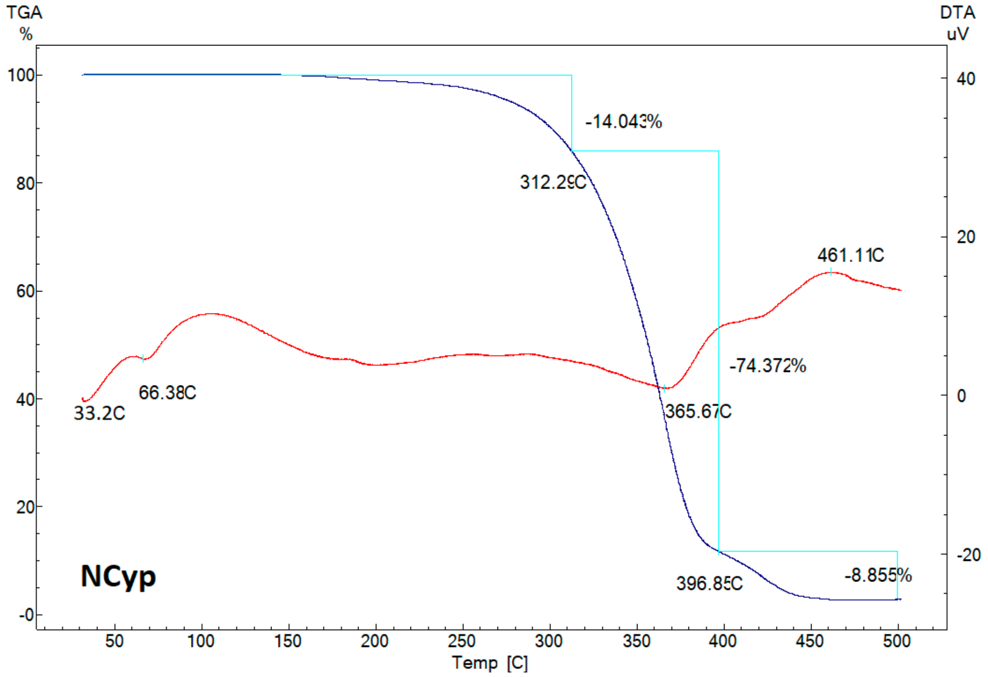
TGA
%
DTA
uV
100
80
60
40
20
-0
40
20
0
-20
-14.043%
312.29C
461.11C
-74.372%
33.2C
66.38C
365.67C
396.85C
-8.855%
NCyp
50
100
150
200
250
300
350
400
450
500
Temp [C]

(c)

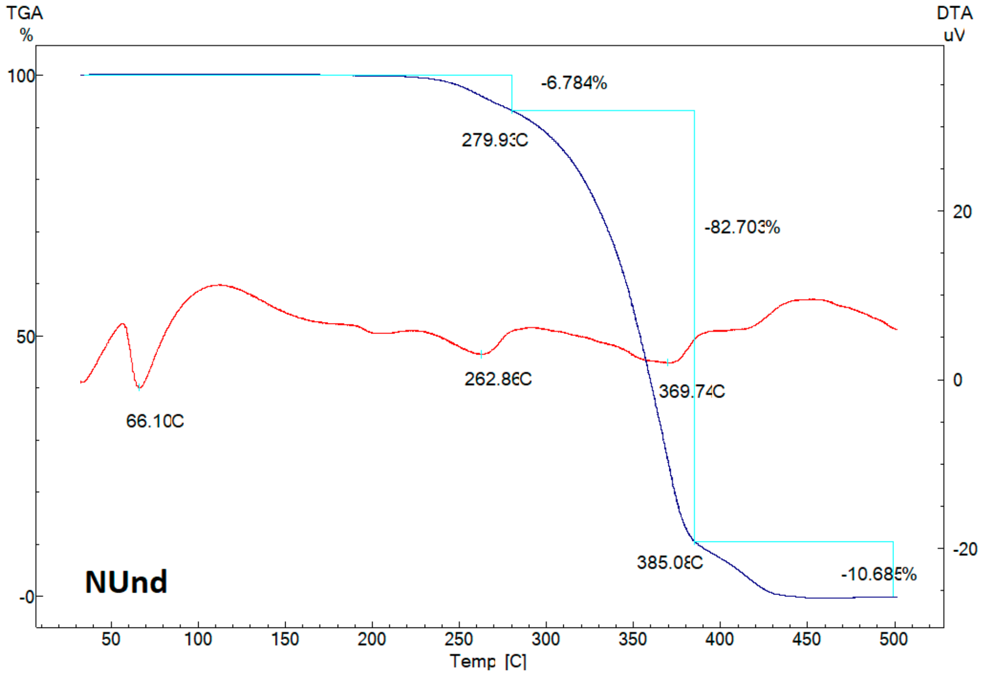

(d)

Figure S5. *Thermal DTA/TG diagrams of analyzed esters*
